# Annual Temperature Reconstruction by Signal Decomposition and Synthesis from Multi-Proxies in Xinjiang, China, from 1850 to 2001

**DOI:** 10.1371/journal.pone.0144210

**Published:** 2015-12-03

**Authors:** Jingyun Zheng, Yang Liu, Zhixin Hao

**Affiliations:** 1 Key Laboratory of Land Surface Pattern and Simulation, Institute of Geographical Sciences and Natural Resources Research, Chinese Academy of Sciences, Beijing, 100101, China; 2 University of Chinese Academy of Sciences, Beijing, 100049, China; Chinese Academy of Sciences, CHINA

## Abstract

We reconstructed the annual temperature anomaly series in Xinjiang during 1850–2001 based on three kinds of proxies, including 17 tree-ring width chronologies, one tree-ring δ^13^C series and two δ^18^O series of ice cores, and instrumental observation data. The low- and high-frequency signal decomposition for the raw temperature proxy data was obtained by a fast Fourier transform filter with a window size of 20 years, which was used to build a good relationship that explained the high variance between the temperature and the proxy data used for the reconstruction. The results showed that for 1850–2001, the temperature during most periods prior to the 1920s was lower than the mean temperature in the 20th century. Remarkable warming occurred in the 20th century at a rate of 0.85°C/100a, which was higher than that during the past 150 years. Two cold periods occurred before the 1870s and around the 1910s, and a relatively warm interval occurred around the 1940s. In addition, the temperature series showed a warming hiatus of approximately 20 years around the 1970s, and a rapid increase since the 1980s.

## Introduction

Long-term regional temperature data are essential for assessing global warming and its impacts on a regional scale over the past century [1]. Recently, several global surface air temperature (SAT) datasets have been constructed with temporal coverage extending back to the 1850s or even earlier [2–5]. Moreover, some studies have focused on the continuous, consistent SAT series of the national average estimation for China during the 20th century [6–10]. However, regular meteorological observations in China started in the 1950s, and prior to the 1950s, only a small amount of instrumental data is available from some eastern stations [11]. Most of the data are non-homogeneous because of inconsistent observational criteria during different years, the relocation of stations, and missing measurements [12, 13]. Therefore, regional temperature series might be reconstructed from proxy data with high time resolution (e.g., tree-rings, ice cores), to extend the datasets to compensate for the deficiencies in the instrument observations, especially in western China (e.g., in Xinjiang, Tibet), where few observations are available prior to the 1950s.

**Fig 1:**
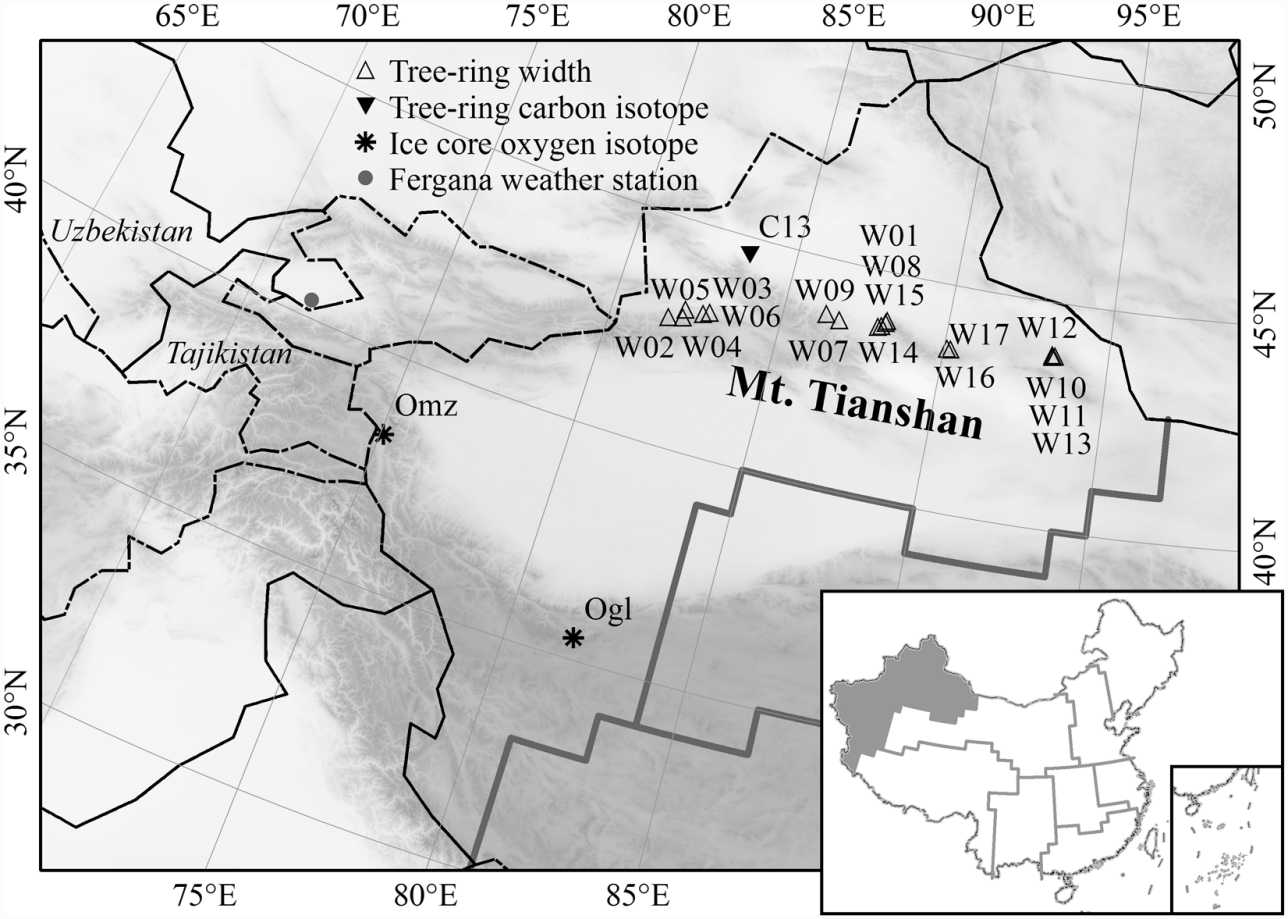
Study area and locations of the proxy data used for the annual temperature reconstruction in Xinjiang. Bottom right: subregions categorized by climate regionalization and the coherence of the temperature change in China (reproduced from Ref. [14]).

Xinjiang is located in northwest frontier of China, the hinterland of the Eurasian continent. Fig 1 shows the study area divided by Wang et al [14] according to China climate regionalization and the coherence of temperature change. There were many studies have focused on reconstructing temperature changes by using a single tree-ring proxy in several subareas in Xinjiang. For example, in the drainage basin of the Jinghe River and the Boertala River in the north of the Tianshan Mountains, summer temperatures for the past 500 years were reconstructed by using tree-ring width chronologies of *Picea schrenkiana* [15, 16]. Tree-ring chronologies of width or maximum density were used to reconstruct the maximum temperature, mean temperature, or minimum temperature over the last few hundred years for Jimsar and Barkol counties in the eastern Tianshan Mountains [17, 18], the Hutubi river basin, the Urumqi riverhead, the Gongnaisi region in the central Tianshan Mountains [19–21], the Yili river valley in the western Tianshan Mountains [22–24], and Altay Mountain in northern Xinjiang [25–26]. However, all of these studies only focused on a certain period during the year (e.g., summer, or April to May, May to September, December to March) and a restricted locality, which is not adequate for representing the annual temperature changes for the whole Xinjiang region.

In the 1990s, Wang et al. reconstructed the homogenous annual temperature series from 1880 to 1996 for ten regions of China, including Xinjiang (Fig 1), based on multi-proxy temperature data including documentary, ice core, and tree-ring data, and the fragmentary instrumental temperature data [14]. Although this dataset was important because it depicted regional temperature changes in China during the last century [13, 27], the authors themselves highlighted several flaws. For example, the limitations of proxy spatial coverage and large uncertainty because of the weak correlation between regional temperature changes and the proxies used to calibrate the reconstruction. Specifically, the mean annual temperature series in Xinjiang was reconstructed based on the δ^18^O series from the Guliya ice core alone, and the correlation coefficient between the δ^18^O series and mean annual temperature in Xinjiang was only 0.305, which indicated the high uncertainty of this reconstruction [14]. Thus, it is necessary to develop a higher-quality reconstruction of the mean annual temperature from the different proxies available, and to use a new methodology.

## Data and Method

### Proxy and instrumental data

Three kinds of proxies, including 17 tree-ring width chronologies, one δ^13^C series from tree-rings, and two δ^18^O series from ice cores, are used for the annual temperature reconstruction of the study area, Xinjiang. The sites of all proxies are shown in Fig 1. All of the 17 tree-ring width chronologies (designated W01–W17 in Fig 1) are located in the Tianshan Mountains in central Xinjiang, and the standard chronology for each site is built from the raw measurements by spline detrending with 50% frequency cutoff at 67% of the curve length to remove tree-age related growth trends in each tree. The tree-ring δ^13^C series (C13 in Fig 1) is from Aibi Lake Valley, north of the Tianshan Mountains [28]. The δ^18^O series from the Guliya ice core (Ogl in Fig 1) [29], and Muztagata (Omz in Fig 1) [30], are obtained in the northwest part of the Tibetan Plateau, but in the southwest of the study area. These proxies are available from the World Data Center for Paleoclimatology (http://www.ncdc.noaa.gov/data-access/paleoclimatology-data/datasets) and 17 of them are available for the entire period from 1850 to 2002. However, the tree-ring width chronology of *Picea schrenkiana* from Baiyang Valley (W01) in the Tianshan Mountains begins in 1867, the δ^18^O series from the Muztagata ice core begins in 1907, and the δ^18^O series from the Guliya ice core ends in 1991. All datasets are published in peer-reviewed international journals and checked by the data quality criteria provided by PAGES (Past Global Changes, http://www.pages.unibe.ch/download/docs/working_groups/2k_network/pages2k-proxy-selection-criteria-Aug2014.pdf), and the reliabilities of the datasets have been discussed in their original studies [28–31]. Information about these proxy data is listed in S1 Table.

The instrumental data used in this study is the gridded dataset of monthly temperature anomalies in China (named SURF_CLI_CHN_TEM_MON_GRID_0.5) for the reference period of 1971–2000, with a 0.5° × 0.5° spatial resolution beginning in January, 1951 [32]. This dataset is developed and updated by the National Climate Center and released by the Chinese Meteorological Administration on the Climate Data Center website (http://cdc.nmic.cn/home.do). Because the study aims to reconstruct temperature anomalies in Xinjiang, the mean of the annual temperature anomaly for all grid points in the study area is calculated for calibration and validation, and the mean of the monthly temperature anomaly for all grid points in the study area is calculated to interpret the significance of the proxy series to temperature change.

### Significance of the proxy series to temperature

To interpret the significance of the proxy series to temperature, we calculate the correlation coefficients between each proxy series and the regional temperature anomalies for spring, summer, autumn and the entire year (Table 1). The result shows that five proxy series (W02, W04, C13, Ogl, Omz) are significantly correlated with the regional annual temperature anomalies, of which the C13 series is the most significant. Moreover, some tree-ring width chronologies are significantly correlated with seasonal temperature rather than annual temperature. Correlation coefficients between proxies and temperature for the first-order difference and low-pass smoothing by 0.1–0.04 Hz (10–25 years) fast Fourier transform filter (FFT) smoothing are also calculated. The results indicate that most tree ring width series contain high- or low-frequency signals of temperature variations, or both (e.g., W04), but W09, W10, W11, W12, and W17 have no significant correlation with temperature. Meanwhile, the tree-ring δ^13^C series mainly contains low-frequency signals because the stable carbon isotope series preserves a greater number of low-frequency temperature variations than high-frequency compared with the tree-ring width chronologies [33]. Therefore, the W01–08, W13-W16, C13, Ogl, and Omz series are retained as the candidate proxies for regression during temperature reconstruction.

**Table 1:** Correlation coefficients between the regional temperature anomalies and each proxy series in Xinjiang (only coefficients that exceeded a 90% significance level are listed). Significance level: *p < 0.1, **p < 0.01, *** *p* < 0.001. To test the significance level of the relationship of FFT smoothing between proxy and temperature, the effective number of degrees of freedom for the correlation is calculated by the Monte Carlo method, as suggested by Yan et al. [34].

	Proxy	Correlation period	Original series	First order difference	10-year FFT smoothing	15-year FFT smoothing	20-year FFT smoothing	25-year FFT smoothing
Annual	W02	1951–2004	0.351***	0.225	0.747***	0.933***	0.997***	0.989***
	W04	1951–2004	0.496***	0.306**	0.834***	0.918***	0.980***	0.983***
	C13	1951–2003	-0.643***	-0.195	-0.840***	-0.828***	-0.809**	-0.787*
	Ogl	1951–1991	0.305*	0.276*	0.612*	0.574	0.639	0.530
	Omz	1951–2000	0.408***	0.117	0.836***	0.873***	0.913***	0.909**
Spring	W01	1951–2004	-0.304**	-0.354***	0.309	0.375	0.516	0.600
	W02	1951–2004	0.227*	0.100	0.692**	0.787**	0.839**	0.799*
	W03	1951–2004	0.255*	0.177	0.568*	0.715**	0.832**	0.812**
	W04	1951–2004	0.263*	0.112	0.665**	0.732**	0.837**	0.813**
	W08	1951–2004	-0.069	-0.258*	0.418	0.413	0.518	0.510
	W13	1951–2002	-0.159	-0.350**	-0.111	-0.265	-0.637	-0.652
	W14	1951–2002	0.016	-0.318**	0.441	0.413	0.535	0.642
	W15	1951–2002	-0.234*	-0.353**	0.513*	0.498	0.779**	0.647
	W16	1951–2002	0.050	-0.277**	0.687**	0.917***	0.906***	0.836*
Summer	W02	1951–2004	0.468***	0.521***	0.675**	0.793***	0.874***	0.846**
	W03	1951–2004	0.172	0.256*	0.415	0.589*	0.761**	0.788*
	W04	1951–2004	0.531***	0.605***	0.694**	0.762**	0.891***	0.869**
	W05	1951–2004	0.157	0.249*	0.297	0.283	0.206	0.140
	W06	1951–2004	-0.221	0.147	-0.507*	-0.503	-0.435	-0.489
	W16	1951–2002	0.285**	0.154	0.720***	0.929***	0.926***	0.940**
	C13	1951–2003	-0.307**	-0.153	-0.381	-0.363	-0.360	-0.321
	Omz	1951–2000	0.193	0.272*	0.317	0.375	0.466	0.453
Autumn	W04	1951–2004	0.287**	0.112	0.742***	0.857***	0.966***	0.973***
	W07^a^	1951–2004	-0.277**	-0.231*	-0.186	0.233	0.581	0.837**
	W13	1951–2002	0.078	0.244*	-0.114	-0.231	-0.583	-0.711
	C13	1951–2003	-0.465***	-0.116	-0.854***	-0.846***	-0.848**	-0.830*
	Ogl	1951–1991	0.381**	0.409***	0.299	0.263	0.750	0.706
	Omz	1951–2000	0.241*	0.055	0.835***	0.893***	0.941***	0.935**

^a^Correlation coefficients between W07 and the temperature from July to October

### Reconstruction method

We use an approach based on multi-scale signal decomposition and synthesis to combine the largest number of low- and high-frequency signals from different proxies and to build a relationship that explains the high variance between the temperature and proxy data for regional temperature reconstruction. This approach is similar to the method used by Moberg et al. [35], which has been used for Northern Hemisphere (NH) temperature reconstruction from low- and high-resolution proxy data.

Let *n* be the number of years for FFT smoothing. The temperature series used for calibration, *T*(*t*), can be decomposed into two series of low- and high-frequencies as

$$T(t)=T{\left(t\right)}_{l/n}+dT(t)$$ (1)

where *dT*(*t*) is the high-frequency series with components higher than 1/*n*, and *T*(*t*)_1/n_ is the component that contains the low-frequency signal, namely the FFT smoothing series obtained by removing the Fourier components with frequencies higher than 1/*n*. Similarly, each proxy series *P*_i_(*t*) can be decomposed as

$$P_{i}(t)=P_{i}{\left(t\right)}_{l/n}+dP_{i}(t)$$ (2)

Next, we establish the calibration equation between the proxy data and temperature to reconstruct the low- and high-frequency temperature signals by using *P*_i_(*t*)_1/n_ and *dP*_i_(*t*), respectively. Finally, the two signals are combined into one reconstruction series. To minimize the multi-linearity effect and avoid variance inflation in the reconstruction, stepwise regression and best subset regression [36] are performed for low- and high-frequency signals, respectively, between the temperature and proxy data. The leave-one-out cross-validation method [37] is then used to calculate predicted *R* ^2^_p_ for low-frequency regression and *r* ^2^_p_ for high-frequency regression. Noted that the tree-ring width in Tianshan Mountains, Xinjiang, is also affected by the temperature during the previous summer and autumn [38]; thus, the high-frequency temperature reconstruction should consider the tree-ring width data from both the current and the subsequent year.

To select the optimal low- and high-frequency signals for temperature reconstruction, signal decomposition is repeated by adjusting the number of years (*n* = 5, 10, 15, 20, 25) for FFT smoothing, and the regression equations with highest *R* ^2^_p_ + *r* ^2^_p_ value are selected as the optimal equations for temperature reconstruction. Noted that the C13 and W04 series have the highest correlation coefficients with annual temperature (Table 1); thus, they are set as the initial variables in the model for stepwise regression. To avoid the multi-linearity effect and variance inflation, subsequent entries are selected that have no significant correlation at *p* = 0.1 with the independent variables in the model. The results show that *R* ^2^_p_ + *r* ^2^_p_ reaches a maximum when *n* = 20, and the FFT smoothing of the instrumental temperature for 1951–2001 shows a significant cold interval around 1970 and the subsequent warming trend. Although *R* ^2^_p_ + *r* ^2^_p_ for *n* ≥ 25 is close to that for *n* = 20, it does not show the cooling interval around 1970. Therefore, the reconstructions for the 20-year FFT smoothing series (low-frequency signals) and the high-frequency series with components higher than 1/20 (high-frequency signals) are performed. The calibration equations are

$$T_{1/20}=-0.603C13_{1/20}+0.305W04_{1/20}+0.287W16_{1/20}+0.150W08_{1/20}$$ (3)

$$\begin{array}{l} dT=0.470dW05-0.385dW16+0.536dW13-0.260dW01-0.261dW06(t+1)-0.264dW07(t+1)\\ +0.958dW14(t+1)-0.947dW15(t+1)-0.281dW16(t+1)+0.501dW01(t+1) \end{array}$$ (4)

where Eq 3 (in format of standardized regression coefficients, same as other equations) is for low-frequency signals and Eq 4 is for high-frequency signals. *T* is temperature, C13, W01, …, W16 are the proxies shown in Table 1, and (*t* + 1) is the tree-ring width for the subsequent year. In Eq 3, *R* ^2^_a_ (explained variance after the degrees of freedom were adjusted, same for *r* ^2^_a_) and *R* ^2^_p_ are 0.994 and 0.992, respectively. In Eq 4, *r* ^2^_a_ and *r* ^2^_p_ are 0.492 and 0.367, respectively.

Because W01 is available only after 1867, the remaining independent variables are used to reconstruct the temperatures during the period 1850–1866, and the calibration equation is

$$\begin{array}{l} dT=0.444dW05-0.237dW08-0.398dW16+0.510dW13-\\ 0.322dW06(t+1)+0.875dW14(t+1)-0.606dW15(t+1)-0.281dW16(t+1) \end{array}$$ (5)

In Eq 5, *r* ^2^_a_ and *r* ^2^_p_ are 0.399 and 0.288, respectively. Therefore, the complete high-frequency reconstruction might be obtained by merging these two results, which have different variances during the calibration period. The standard deviations of the predicted series for the calibration period (1951–2001) derived from Eqs 4 and 5 are 0.309 (s1) and 0.282 (s2), respectively, so the temperature anomalies from 1850 to 1866 resulting from Eq 5 must be adjusted by multiplying a value of s1/s2. Finally, the temperature reconstruction including low- and high-frequencies is synthesized according to Eq 1. All the regression analysis in this study is performed by MINITAB software, the uncertainty interval for the low- and high-frequency reconstruction is set as twice the standard error of prediction (95% confidence level), and the sum of them is set as the uncertainty interval for the synthesized reconstruction.

In addition, temperature reconstruction from proxy data using the best subset regression (traditional method) without signal decomposition is also conducted for comparison. The complete result is calibrated and merged from following equations because some proxy data were not available for a certain period.

$$\begin{array}{l} T=0.39W03+0.46W13-0.38W16-0.54C13+0.240mz+\\ 0.24W01(t+1)-0.40W06(t+1)-0.22W13(t+1)+0.62W14(t+1)-\\ 0.46W15(t+1) \end{array}$$ (6)

$$\begin{array}{l} T=0.37W03+0.41W13-0.29W16-0.63C13+0.32W01(t+1)\\ +0.19W03(t+1)-0.52W06(t+1)-0.28W13(t+1)+0.46w14(t+1)-\\ 0.42W15(t+1) \end{array}$$ (7)

$$\begin{array}{l} T=0.36W03+0.46W13-0.32W16-0.60C13+0.16W04(t+1)-\\ 0.42W06(t+1)-0.25W13(t+1)+0.47W14(t+1)-0.19W15(t+1) \end{array}$$ (8)

Eqs 6–8 are used for the reconstructions for 1907–2000, 1866–1906, and 1850–1865, respectively. In Eq 6, *R* ^2^_a_ = 0.58, *R* ^2^_p_ = 0.49. In Eq 7, *R* ^2^_a_ = 0.56 and *R* ^2^_p_ = 0.46; and in Eq 8, *R* ^2^_a_ = 0.55 and *R* ^2^_p_ = 0.45. Moreover, the ensemble empirical mode decomposition (EEMD) method [39] is applied to compare the difference between reconstructed results from signal decomposition and the traditional method.

## Results and Discussion

**Fig 2:**
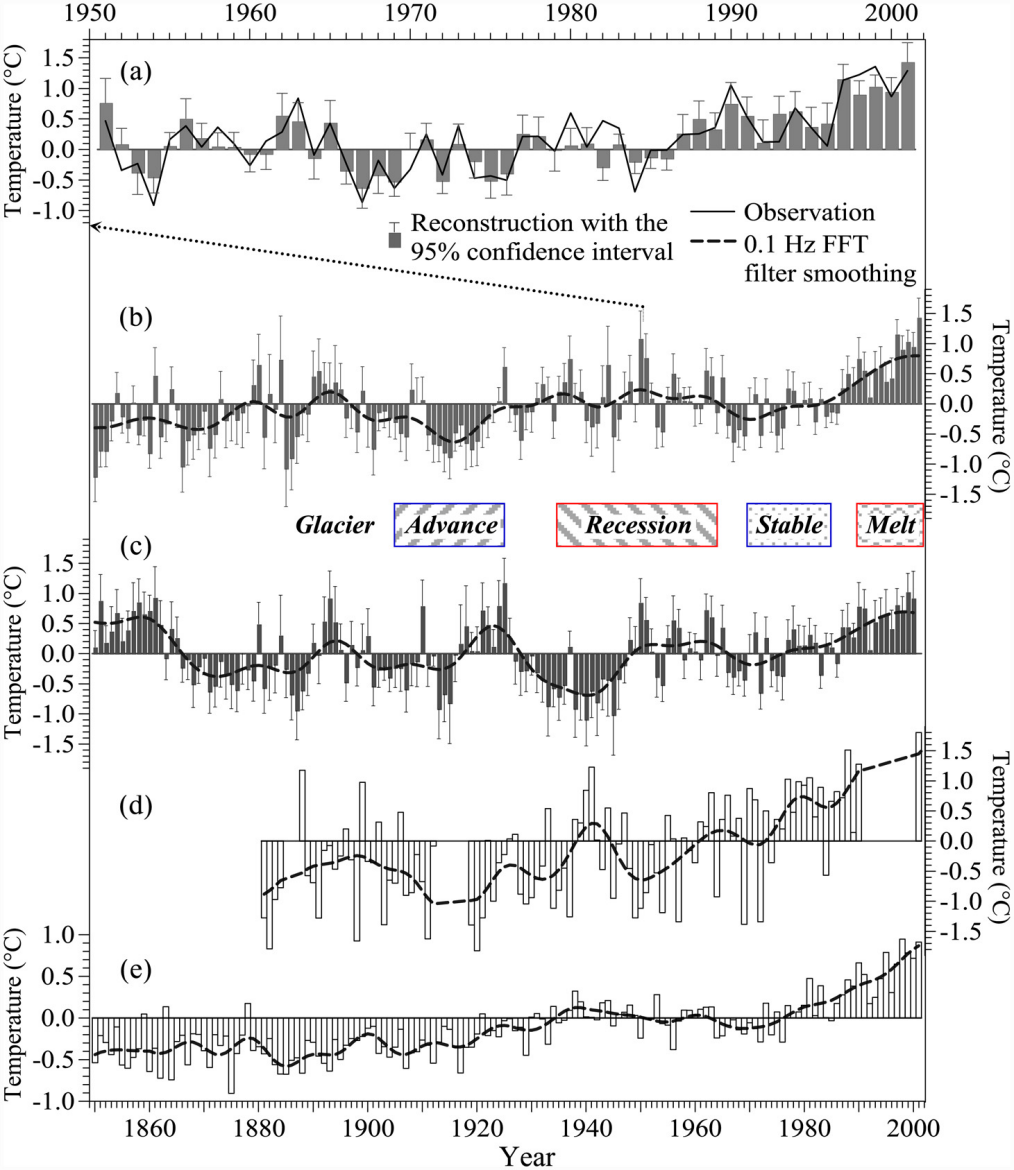
Reconstruction of annual temperature anomalies (with a reference period of 1901–2000; the same for the other series) in Xinjiang during 1850–2001 and comparison with other data. (a) Comparison of the reconstruction and observation for 1951–2001. (b) Annual temperature anomalies with a 95% confidence interval reconstructed by the signal decomposition and synthesis method. (c) Annual temperature anomalies with a 95% confidence interval reconstructed by traditional method. (d) Observed temperature anomalies at Fergana (40.37°N, 71.75°E), Republic of Uzbekistan for 1881–2001 (data from http://climexp.knmi.nl/). (e) NH land air temperature anomalies for 1850–2001 from the Climatic Research Unit, University of East Anglia (http://www.cru.uea.ac.uk/cru/data/temperature/CRUTEM4v-nh.dat). The box between panel (b) and (c) shows the approximate time intervals for glacial activity in the Tianshan Mountains and the other western China highlands during the 20th century [41–43].

Fig 2 shows the reconstructed series of the annual temperature anomaly and its 95% confidence interval in Xinjiang for 1850–2001 with other series for comparison. The comparison (Fig 2a) of the reconstructed and observed annual mean temperatures for 1951–2001 shows that the reconstruction captures observed temperature change well, and the explained variance (EV) is 0.763. The reconstruction (Fig 2b) shows that the temperature in Xinjiang increased gradually at a rate of 0.48°C/100a with significant annual and decadal variations during the period 1850–2001. Two cold periods occur before the 1870s and around the 1910s, and a relatively warm interval occurs around the 1940s, followed by a 20-year warming hiatus around the 1970s and rapid warming since the 1980s. The temperature during most years before the 1920s is lower than the mean for 1901–2000, and the warming trend is visible during the 20th century with a rate of 0.85°C/100a. This rate of increase is similar to that in Tajikistan (0.96°C/100a), located to the west of Xinjiang, which was derived from temperature observations [40]. Comparison shows that the reconstruction for Xinjiang (Fig 2b) and the observed temperature anomalies (Fig 2d) at Fergana (40.37°N, 71.75°E), Republic of Uzbekistan, for 1881–2001, both have similar decadal variation, with the coldest period around the 1910s, a relatively warm interval around the 1940s, a warming hiatus around the 1970s, and rapid warming since the 1980s. Our reconstruction is also consistent with the trend and decadal variations in NH land air temperature (Fig 2e) for 1850–2001.

Moreover, comparing the reconstruction (Fig 2b) with glacial fluctuations in the Tianshan Mountains and the other western China highlands during the 20th century indicates that the inter-decadal temperature variation in Xinjiang coincides with the intervals of glacier advance and recession. For example, during the past 150 years, the coldest period in Xinjiang started at the end of the 19th century and lasted for more than 20 years, followed by the advance of Glacier No.1 in the Tianshan Mountains with the formation around 1910 of a terminal moraine, which was about 280 m from the terminal moraine in the 1980s, as measured by lichenometric dating [41–42]. Meanwhile, most of the glaciers in the highlands of western China, especially in the Qinghai-Tibetan Plateau, all advanced from the 1900s to the 1920s [43]. Subsequently, a relatively warm period in Xinjiang from the 1930s to the 1960s corresponds to an interval of considerable glacier recession over western China that occurred from the 1940s to the 1960s [43]. Moreover, the warming hiatus from 1964 to 1984 in Xinjiang coincided with the intervals from the 1970s to the 1980s when most glaciers remained stable. The rapid warming in Xinjiang in the late 20th century agreed well with a melting period for most glaciers in western China [27, 43].

Comparison of Fig 2b and 2c reveals a significant difference between the two reconstruction series calibrated by the two methods. The reconstruction (Fig 2b) calibrated by signal decomposition and synthesis method (new method in this study) shows an increasing trend with annual and decadal variations for 1850–2001. The significant characteristics of rapid warming from the 1900s to the 1940s, the cold period before the 1870s, and warm interval around the 1940s, are similar to the temperature changes in nearby regions (Fergana, Uzbekistan, Fig 2d), and also on a large scale (NH in Fig 2e). However, the reconstruction (Fig 2c) calibrated by the traditional method does not show the increasing trend in temperature during 1850–2001, which is not consistent with the characteristics of the temperature changes in the nearby regions and NH, and contains warm intervals before the 1870s and around the 1920s and a cold interval around the 1940s. Specifically, the cold interval around the 1940s even contradicts the glacial recession from the 1940s to the 1960s in western China (including the Tianshan Mountains) [42–43]. This comparison demonstrates that the reconstruction calibrated by the new method is more reasonable and reliable than that by the traditional method.

**Fig 3:**
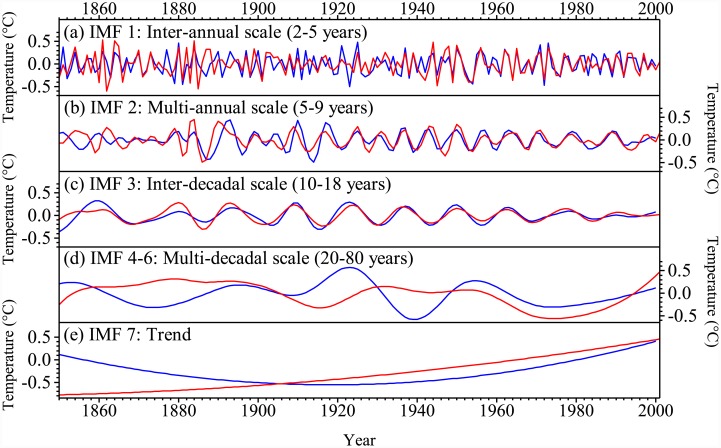
Comparison of IMFs at different scales derived by EEMD (red line for reconstruction by the new method, blue line for that by the traditional method).

In addition, Fig 3 compares the intrinsic mode function (IMF) for the different frequency domains derived by EEMD and their EVs (Table 2) for the reconstructions using the new method and traditional method. It shows that both reconstructions capture a similar temperature variability signal and EV at the frequency higher than 1/20 with IMF1, IMF2, and IMF3 for inter-annual (Fig 3a), multi-annual (Fig 3b) and inter-decadal (Fig 3c) scales. However, in the domain lower than the frequency of 1/20, the sum of IMF4, IMF5, and IMF6 from two reconstructions show similar phases of change mostly at multi-decadal scales (Fig 3d) with different variability, which show a total EV of 16.4% for the new method and 32.3% for the traditional method. IMF7 from the results reconstructed by the new method shows a linear trend with an EV of 24.5%, whereas the results reconstructed by the traditional method show a parabolic trend with an EV of 11.1% (Fig 3e). The EVs for the reconstruction by the new method are similar to those of observations on multi-decadal scale and the trend. However, the reconstruction by the traditional method overestimates the temperature variance on multi-decadal scale, but underestimates that for the trend, which leads to the evident cold signal around the 1940s (Fig 3d).

**Table 2:** The EVs of different frequency domains for the temperature observation and reconstructions by the new and traditional methods.

Scale	Temperature observation	Reconstruction by the new method	Reconstruction by the traditional method
Annual-decadal	0.580	0.591	0.566
Multi-decadal	0.197	0.164	0.323
Trend	0.223	0.245	0.111

Tree-ring width chronology is a principal proxy for annual temperature reconstruction on a regional and large spatial scale because of its advantages of accurate dating and high time resolution [44]. However, tree-ring width chronology usually has a weak, low-frequency variability, especially on a centennial time scale, which may be caused by the ability of trees to adapt to moderate and slow climate change and local environmental changes, especially removing the tree-age related growth trends to build standard chronology [45]. This limitation may be the reason that climate reconstruction illustrates the low-frequency climate change signal and trend in long-term changes poorly, which is one of the important challenges in dendroclimatology. To retain more low-frequency variability during the standardization of width chronology, dendroclimatologists have developed a new approach called “regional curve standardization” (RCS) instead of the “classic” standardization approach, which removes the age dependence from each individual tree-ring record [46]. However, the RCS approach is based on the assumption that the age dependence of all tree-ring width records for the same species sampled in a climatically homogeneous geographic region should be described well by a single mean curve that may overestimate the low-frequency variability, especially in western China [45]. Therefore, there is an urgent need to improve the calibration method for climate reconstruction to capture the low-frequency signal retained in the standard tree-ring width chronology after the tree growth is detrended. Recently, Shi et al. adopted a signal decomposition method to facilitate the assimilation of proxy data with different temporal resolutions for reconstructing China and Asia temperature variations in the frequency domain lower than 10-year [47–48]. Our reconstruction in this study uses a new method to calibrate the temperature and the proxy data via signal decomposition and synthesis of the low- and high-frequencies, which avoids the errors caused by inconsistent phases of climate fluctuation at different frequencies. This new method is not only able to retain the inter-annual temperature variability signal from the tree-ring data, but also able to capture the decadal temperature variation and long-term changing trends from the multi-proxies. Thus, this study may provide a useful method for climate reconstruction using tree-ring data.

## Conclusion

We reconstructed the annual temperature anomaly in Xinjiang during 1850–2001 using low- and high-frequency signal decomposition method and synthesis from tree-ring width chronologies, tree-ring δ^13^C series, and ice core δ^18^O series. We investigated the advantages of the calibration method for temperature reconstruction by multi-scale signal decomposition and synthesis from multi-proxies, and analyzed the characteristics of temperature changes in Xinjiang during the period 1850–2001. Our main conclusions can be summarized as follows.

(1) The temperature in Xinjiang increased during the period 1850–2001 with significant decadal variations, where two cold periods occurred before the 1870s and around the 1910s, but a relatively warm interval occurred around the 1940s. A 20-year warming hiatus occurred around the 1970s, and rapid warming has prevailed since the 1980s. The temperatures for most years before the 1920s were lower than the mean for 1901–2000, and remarkable warming occurred during the 20th century, at a rate of 0.85°C/100a.

(2) Compared with the reconstruction calibrated by direct regression between temperature and multi-proxy series, the reconstruction calibrated by the method of multi-scale signal decomposition and synthesis from multi-proxies is more reasonable and more reliable. Our new method can recover the inter-annual temperature variability signal from the tree-ring data and capture the decadal temperature variations and long-term changing trend from the multi-proxies, which are important for tracking global warming during the 20th century.

## Supporting Information

S1 Table Proxy data for 20 sites used in the study. (DOC)
