# Supplementary material for: Annual Temperature Reconstruction by Signal Decomposition and Synthesis from Multi-Proxies in Xinjiang, China, from 1850 to 2001
Source: PLoS One. 2015 Dec 3;10(12):e0144210. doi: 10.1371/journal.pone.0144210 (PMC4669149; doi:10.1371/journal.pone.0144210)
Supplement: S1 Table — (DOC) [file pone.0144210.s001.doc]

**Supporting Information**

**S1 Table. Proxy data for 20 sites used in the study**

| Proxy type | No. | Site | Latitude | Longitude | Altitude | Species | Start/end years |
| --- | --- | --- | --- | --- | --- | --- | --- |
| Tree-ring width | W01 | Baiyang Valley | 43.93°N | 88.15°E | 1815m | *Picea schrenkiana F.* | 1867-2004 |
|  | W02 | Big Kushitai | 42.88°N | 82.13°E | 2763m | *Picea schrenkiana F.* | 1690-2004 |
|  | W03 | Jialepake | 43.07°N | 82.15°E | 2308m | *Picea schrenkiana F.* | 1605-2004 |
|  | W04 | Kengbulake | 42.80°N | 81.72°E | 2593m | *Picea schrenkiana F.* | 1686-2004 |
|  | W05 | Kuerdening | 43.15°N | 82.87°E | 1499m | *Picea schrenkiana F.* | 1634-2004 |
|  | W06 | Qiaxi | 43.08°N | 82.68°E | 1710m | *Picea schrenkiana F.* | 1721-2004 |
|  | W07 | Shitizi East Valley | 43.72°N | 86.72°E | 1865m | *Picea schrenkiana F.* | 1785-2004 |
|  | W08 | Tian Chi | 43.88°N | 88.12°E | 1913m | *Picea schrenkiana F.* | 1694-2004 |
|  | W09 | Xiaoxi Valley | 43.78°N | 86.30°E | 1878m | *Picea schrenkiana F.* | 1706-2004 |
|  | W10 | Xinjiaing BLKA | 43.85°N | 93.30°E | 2810m | *Larix sibirica L.* | 1571-2002 |
|  | W11 | Xinjiaing BLKB | 43.83°N | 93.38°E | 2840m | *Larix sibirica L.* | 1608-2002 |
|  | W12 | Xinjiaing BLKC | 43.82°N | 93.33°E | 2480m | *Larix sibirica L.* | 1790-2002 |
|  | W13 | Xinjiaing BLKD | 43.82°N | 93.30°E | 2380m | *Larix sibirica L.* | 1807-2002 |
|  | W14 | Xinjiaing MIQA | 43.77°N | 87.92°E | 1970m | *Picea schrenkiana F.* | 1653-2002 |
|  | W15 | Xinjiaing MIQB | 43.80°N | 88.02°E | 2080m | *Picea schrenkiana F.* | 1653-2002 |
|  | W16 | Xinjiaing MULA | 43.60°N | 90.22°E | 2250m | *Picea tienschanica R.* | 1715-2002 |
|  | W17 | Xinjiaing MULB | 43.60°N | 90.10°E | 2170m | *Picea tienschanica R.* | 1829-2002 |
| δ^13^C from tree-ring | C13 | Aibi Lake | 44.62°N | 83.57°E | 280m | *Populus Euphratica O.* | 1835-2005 |
| δ^18^O from ice cores | Ogl | Guliya Ice Cap | 35.28°N | 81.48°E | 6200m |  | 1570-1991 |
|  | Omz | Muztagata Glacier | 38.28°N | 75.10°E | 7010m |  | 1907-2002 |
